# Limits of bacterial osmoadaptation during planktonic and biofilm growth: a step toward effective biofouling control

**DOI:** 10.1128/aem.02411-25

**Published:** 2026-04-21

**Authors:** Jan Struckmann Poulsen, Arya Van Alin, Peter Bundgaard Larsen, Freja Marie Nordby Haarder, Rikke Louise Meyer, Klaus Koren, Kasper Urup Kjeldsen

**Affiliations:** 1 Department of Biology, Section for Microbiology, Aarhus University 683568, Aarhus, Denmark; 2 Interdisciplinary Nanoscience Centre (iNANO), Aarhus University 530037, Aarhus, Denmark; Indiana University Bloomington 1771, Bloomington, Indiana, USA

**Keywords:** salinity fluctuation, adaptation, biofilm, biofouling, cell-specific oxygen consumption

## Abstract

Salinity is a key parameter for bacterial survival and growth. Halophilic and halotolerant bacteria can adapt to elevated salinity, but the energetic demands of osmoadaptation increase under fluctuating salt concentrations, potentially constraining growth and persistence. A new concept in reverse osmosis (RO) filtration is batch operation with oscillating rather than constantly high brine salinity. We hypothesize that fluctuating salinity can diminish biofouling in such RO systems. To test this hypothesis, we examined the survival and activity of *Aliivibrio fischeri* and *Pseudomonas fluorescens* under fluctuating salinities in planktonic and biofilm cultures as representative of halophilic and halotolerant species, respectively, and common members of biofouling communities. At 28°C, *P. fluorescens* grew at 0%–6% salinity with fastest growth rate at 0%–1%. At 7%–10% salinity, *P. fluorescens* remained viable but did not grow. At 22°C, *A. fischeri* grew at 0.5%–7% salinity, with fastest growth rate at 2%–3%, but unlike *P. fluorescens*, it lost viability outside this growth range. Cultures did not respire at salinities that did not support growth, suggesting that survival under such salt stress does not depend on high metabolic activity. Furthermore, cell-specific aerobic respiration rates in *A. fischeri* correlated with growth rate but not osmotic stress. Biofilm formation did not enhance the osmotic stress tolerance of the two bacteria. Our results indicate that high constant salinity favors the halophilic *A. fischeri* over the halotolerant *P. fluorescens*, but oscillating salinity (e.g., 0%–7%) favors neither. Oscillating salinity may, therefore, offer a new mechanism for controlling microbial growth that circumvents community adaptation to environmental conditions.

**IMPORTANCE:**

Reverse osmosis filtration is a widely used technology to address the scarcity of clean freshwater. However, the efficiency of reverse osmosis systems is challenged by microbial biofouling, as microbial communities adapt to the environmental conditions within the system and form biofilms on the membranes. This study investigated the impact of fluctuating salinity on the growth and survival of halophilic and halotolerant bacteria. The findings suggest that oscillating salinity disrupts the growth and viability of both types of bacteria, in both planktonic cultures and biofilms. The study, thus, supports the hypothesis that fluctuating salinity in reverse osmosis systems could reduce biofouling by impeding microbial adaptation to salinity. This represents a promising new strategy for microbial control in reverse osmosis systems, potentially enhancing performance by minimizing biofouling through an environmentally friendly approach.

## INTRODUCTION

Bacteria populate virtually every conceivable environmental niche on Earth. They range from extremophiles in the planet’s harshest environments to those that have colonized the human body. Their ability to thrive in diverse environments is due to effective mechanisms for sensing and responding to external changes. Salinity is a crucial environmental factor that structures the composition of bacterial communities (1). Different bacteria can tolerate varying ranges of salinities: non-halophilic species show impaired growth at salt concentrations above a few percent, whereas halotolerant species can grow across a broader range of salinities, and halophiles and extreme halophiles require elevated salt concentrations to thrive (2). Bacterial cells must regulate their internal environment to maintain osmotic balance, ensuring proper turgor pressure across the cell wall, with the cytoplasm and periplasm typically being isoosmotic (3, 4). Most bacteria respond to hyperosmotic shock by an initial rapid uptake of potassium ions, followed by the synthesis or uptake of organic anions to counterbalance the charge (4, 5). However, the upper salinity limit that can be mitigated by potassium ion accumulation is around 3% NaCl (6); beyond this, high intracellular potassium concentrations can interfere with cellular processes, necessitating a shift to the secondary osmoregulation response. In most halotolerant and halophilic bacteria, this second long-term response involves maintaining a lower intracellular salt concentration and balancing the cell’s osmolarity by accumulation of compatible solutes, which do not interfere with cellular processes even at high concentrations (4, 6). In contrast to this “salt out” strategy, certain extreme halophilic bacteria and archaea utilize a “salt in” strategy where cells accumulate high concentrations of KCl to maintain osmotic balance in hypersaline environments (2).

Synthesis or uptake of compatible solutes and their fast export upon hyper- or hypoosmotic shocks are energetically expensive processes (7), but the salt out strategy provides a stable internal environment, allowing halotolerant and halophilic microorganisms to osmoregulate and thrive across a wide range of salinities (8). Due to the energetic cost of osmoregulation, microbial cells increase their maintenance energy requirement when exposed to elevated salinities as documented by studies of both pure cultures (9–11) and environmental communities (12). If not compensated by increased metabolic activity or growth yield (11, 13), this will slow down or diminish the rate of growth. Exposure to continuously fluctuating salinity is energetically challenging for cells because osmoregulation under such conditions would demand repeated accumulation and export of compatible solutes. This is likely pronounced under environmental conditions where nutrient availability is limited and where exported compatible solutes are lost and not available for reuptake. In agreement, the growth rate of yeast cells negatively correlates with the frequency of moderate osmotic stress exposure, and the effect is enhanced by substrate starvation (14–16). Furthermore, salinity fluctuations negatively affect the nitrification and denitrification activity in estuarine sediments (17, 18) and the carbon cycling in anaerobic reactors and soils (19, 20).

We hypothesize that fluctuating salinity can serve as an environmentally friendly strategy to limit unwanted bacterial activity and growth in industrial settings, such as reverse osmosis (RO) water treatment systems. In RO systems, biofilm formation by halotolerant bacteria on the brine side of membranes is a major problem (21). In continuous mode operation, the brine salinity is constant over time. However, batch mode operation is gaining interest because recent studies show a dramatically lower level of membrane fouling in these systems where the brine salinity oscillates (22). We hypothesize that batch mode RO filtration is less susceptible to biofouling because this salinity oscillation limits bacterial growth due to the high energetic expenditure needed for repeated osmoadaptation to changing salinity.

To test this hypothesis, we investigated the effects of hypoosmotic, hyperosmotic, and fluctuating salinity conditions on the survival and respiratory activity of the bacterial species *Pseudomonas fluorescens* and *Aliivibrio fischeri*. These two species each have a well-characterized salinity growth response and are representative of halotolerant and halophilic microorganisms, respectively, both employing a salt out strategy for osmoregulation (23–25). Moreover, both species are known members of biofouling communities in RO systems and are frequently used as model organisms for studying biofouling (26–29). We conducted experiments under both nutrient-depleted and nutrient-rich conditions to determine whether osmoregulation depends on substrate availability and high metabolic activity. Additionally, our experiments included both planktonic and biofilm cultures of the two species. Some microorganisms produce extracellular polymeric substances, which are key components of the biofilm matrix, and form biofilms in response to osmotic stress (30–32) and can, thereby, enhance their salinity tolerance (33). However, although the biofilm mode of growth is generally considered to protect against adverse environmental stress, its protective function against osmotic stress appears complex. The marine bacterium *Vibrio parahaemolyticus* forms biofilms when challenged by hypoosmotic salinity stress (34), while other marine *Vibrio* species form biofilms in response to hyperosmotic stress (30), and in *Pseudomonas aeruginosa*, osmotic stress limits biofilm formation (35). Furthermore, the physical structure of biofilms is dependent on the ionic strength of their environment (36), and their protective effect may, therefore, change in response to the salinity. It remains, therefore, unclear if biofilms show enhanced tolerance to hypo- and hyperosmotic salinity extremes as compared to planktonic cultures.

Overall, our study aimed to determine whether salinity fluctuations more effectively limit bacterial activity and survival compared to constant salinity-induced osmotic stress and thereby gain insight into the potential of using fluctuating salinity to control biofouling in engineered systems, such as water treatment plants using RO filtration.

## MATERIALS AND METHODS

### Strains and media

The bacterial species *Pseudomonas fluorescens* DSM 50090 and *Aliivibrio fischeri* DSM 507 were obtained from DSMZ (German Collection of Microorganisms and Cell Cultures, Germany) and were routinely cultured aerobically in liquid and on agar-solidified R2A medium (LAB203, NeoGen). To replicate the ionic composition of natural saline environments, the salinity of growth media and saline solutions was adjusted using concentrated solutions of Synthetic Sea Salt (Himedia, Germany). The salinities of the latter were calibrated with a handheld refractometer (HC-CARGO, Denmark).

### Measurement of planktonic growth rates

Growth experiments were conducted in 200 µL volumes in 96-well plates at 28°C or 22°C for *P. fluorescens* and *A. fischeri* cultures, respectively. The growth medium was inoculated with 2 µL overnight cultures to achieve a starting optical density at 600 nm (OD_600_) of 0.01 and incubated at room temperature with 200 rpm orbital shaking. Bacterial growth was monitored by OD_600_ measurements every 15 min for 48 h in a CLARIOstar Plus plate reader (BMG Labtech, Germany). Data were processed using the R package GrowthCurver (37).

### Influence of salinity on viability of planktonic cells

The survival of *P. fluorescens* and *A. fischeri* cells during exposure to various synthetic sea salt concentrations was assessed by counting colony-forming units (CFUs) on R2A agar. For these experiments, planktonic overnight cultures of *P. fluorescens* were grown aerobically in 20 mL R2A broth without salt amendment at 28°C and 150 rpm shaking. *A. fischeri* was also grown aerobically as overnight cultures in 20 mL R2A broth at 150 rpm shaking, but with 2% (wt/vol) synthetic sea salt at 20°C. Aliquots of 10 μL from the overnight stationary phase cultures were transferred into 10 mL of sterile salt solution (either in R2A medium or Milli-Q water) in 50 mL Falcon tubes. Synthetic sea salt concentrations of 0.5%, 4%, 7%, and 10% (wt/vol) were tested. The tubes were incubated at 28°C for *P. fluorescens* and 22°C for *A. fischeri* with 150 rpm shaking for 24 h. Samples for CFU counts were collected at the start and after 3, 6, and 24 h of incubation. The experimental design included three replicates for each salt concentration and exposure time, along with one negative control (medium without cells) for each exposure time. CFU counts were performed by the drop plate method (38). Tenfold dilution series (10-, 100-, 1,000-, and 10,000-fold) were prepared. Dilutions for *P. fluorescens* were made in sterile Milli-Q water, while dilutions for *A. fischeri* were prepared in sterile 2% (wt/vol) synthetic sea salt solution. Twenty microliters from each dilution was plated on R2A agar with appropriate salinity levels and incubated at 28°C for *P. fluorescens* and 22°C for *A. fischeri* for 19–26 h before colony counting.

### Synthesis of oxygen-sensing nanoparticles

Oxygen-sensing nanoparticles were synthesized and used for determining oxygen concentrations in planktonic and biofilm cultures. The particles are compatible with concurrent determination of OD_600_ values in a 96-well-plate experimental setup. The synthesis was based on the oxygen-sensing component platinum(II) meso-tetrakis(pentafluorophenyl)-porphyrin (PtTFPP) (Frontier Specialty Chemicals, UT), the antenna dye Macrolex Fluorescent Yellow 10GN (MY) (Lanxess, Germany), and poly(styrene-co-maleic anhydride) (PSMA) (Biosynth, Switzerland), all of which were dissolved in tetrahydrofuran (THF) (Sigma-Aldrich, Denmark) with constant stirring. The concentrations were maintained at a 1:1 ratio of PtTFPP to MY (1.5 mg of each dye per g of PSMA polymer) and a starting concentration of PSMA in the THF solution of 2 mg mL^−1^. Nanoparticles were generated by flash nanoprecipitation (39). Oxygen-sensing components in THF solution were mixed with Milli-Q water as an antisolvent using a confined impinging jet mixer. The solvent was removed from the nanoparticle suspension by air bubbling for approximately 4 h at room temperature, followed by evaporation at approximately 70°C for 8–12 h. This resulted in an aqueous suspension of sensor nanoparticles. The final nanoparticle concentration (1.4 mg mL^−1^) was determined by evaporating the remaining liquid from 1 mL of the nanoparticle suspension and measuring its weight. Nanoparticle size was characterized by dynamic light scattering using an ALV system (ALV GmbH, Germany) operating at a wavelength of 633 nm, with scattered light detected at an angle of 90^o^. Data analysis was conducted with the ALV Correlator 3.0 software (Fig. S1).

### Cell-specific oxygen consumption during osmotic stress

The effect of constant osmotic stress on the cell-specific oxygen consumption rate (csOCR) was determined for *A. fischeri* and *P. fluorescens* planktonic exponential-phase cultures. Oxygen concentrations and OD_600_ were measured concurrently using the CLARIOstar Plus plate reader. The experiments were made in 96-well plates as described above, where each well contained a volume of 194 µL R2A medium with different synthetic sea salt concentrations and 4 µL oxygen-sensing nanoparticle suspension and was inoculated with 2 µL overnight culture. The plate reader was configured to scan the microplate wells every 15 min for a predetermined number of cycles. For all oxygen measurements, the following settings were applied: time-resolved fluorescence method, plate mode with bottom optics, an excitation wavelength of 330 ± 40 nm, a dichroic long-pass filter, and an emission wavelength of 645 ± 20 nm. Two multichromatic settings were used: multichromatic 1 with an integration start at 10 μs and an integration time of 30 μs and multichromatic 2 with an integration start at 50 μs and an integration time of 30 μs, both using the same excitation, dichroic, and emission settings as described above. This oxygen protocol yielded two measurements, which were used to determine the luminescence lifetime of the indicator dye following the rapid lifetime determination method, as previously described (40, 41).

A multipoint calibration was conducted using the Stern-Volmer relationship that relates the change in luminescence intensity (*I*) and the luminescence lifetime (τ) to the O_2_ concentration (42). The obtained calibration curve and the respective two-site model fit are shown in Fig. S2. All experiments included three uninoculated wells filled with growth medium and oxygen-sensing nanoparticles as oxic negative controls for growth. Similarly, three uninoculated wells containing sodium sulfite (10 g L^−1^) as O_2_ scavenger served as anoxic negative controls. The oxic and anoxic negative controls were checked with the oxygen calibration curve to ensure consistent data quality.

For the combined determination of oxygen consumption rate and microbial growth, the OD_600_ was first measured. Shaking of the 96-well plate was arrested during these measurements and 10 s prior to minimize liquid movement in the wells. Next, the oxygen concentration was measured, followed by an 8-min waiting period without shaking. Finally, the oxygen concentration was measured again. The three measurements were executed every 15th minute. During the idle time between protocol executions, shaking was turned on at 200 rpm to prevent full oxygen depletion of the liquid. Oxygen consumption rate was calculated by subtracting the second oxygen measurement from the first, indicating the quantity of oxygen utilized during the interval between the two readings. Microbial growth was assessed by monitoring the change in OD_600_ values over time. The OD_600_ measurements were correlated with viable cell numbers by an OD_600_-CFU number standard curve determined in a separate experiment (Fig. S3).

It should be noted that the calculated csOCR is an approximation, because the oxygen diffusion into cultures during the rate measurements is not accounted for. This is not expected to significantly impact the results, but it is mentioned here for clarity and completeness.

### Influence of constant and fluctuating salinity on the respiratory activity of biofilms

Overnight planktonic cultures of *P. fluorescens* and *A. fischeri* were prepared as described above. The following day, biofilms were initiated by transferring 158 μL of sterile R2A medium with no salt amendment (*P. fluorescens*) or containing 2% (wt/vol) synthetic sea salt (*A. fischeri*) to a 96-well Nunclon Delta Surface plate with Nunc Immuno TSP peg lids (Thermo Fisher Scientific, Denmark). Then, wells were inoculated with 2 μL of the overnight cultures. The plate was incubated statically at 22°C for 2 h to allow cell attachment to the peg lid. Subsequently, the peg lid was moved to a new plate containing 160 μL of sterile R2A medium with 0% and 2% (wt/vol) synthetic sea salt for *P. fluorescens* and *A. fischeri*, respectively, and incubated statically for 2 days at 22°C to facilitate biofilm formation on the pegs. All biofilm experiments were conducted with such freshly grown peg lid biofilms. For the constant salinity experiment, a 96-well plate was prepared with R2A media with different salinities ranging from 0% to 10% (wt/vol). The fluctuation experiment was made by successively transferring the peg-lid biofilms to 96-well plate wells with 160 µL Milli-Q water or pure synthetic sea salt of 10% (wt/vol) salinity, incubating the biofilm for 20 min at each salinity with a washing step in between.

*P. fluorescens* biofilms were washed between transfers by briefly dipping them in pure Milli-Q water. This treatment was repeated three times, resulting in three times fluctuation between 0% and 10% (wt/vol) salinity. As control experiments, biofilms were also exposed to three times 0% or 10% (wt/vol) salinity with no washing steps in between. A similar set of experiments was conducted for *A. fischeri* biofilms; here, the washing solution contained 2% (wt/vol) synthetic sea salt. The control experiments included three times exposure to 0%, 2%, or 10% (wt/vol) salinity with no washing steps in between. Finally, the oxygen consumption rate of the biofilms was determined as described above by transferring the biofilms to 96-well plates with fresh R2A medium of optimum salinity containing oxygen-sensing nanoparticles. Each treatment was replicated eight times, and negative controls with no treatment (optimum salinity) and eight uninoculated blanks were included in the experiments.

## RESULTS

### *P. fluorescens* is halotolerant, and *A. fischeri* is halophilic

**Fig 1:**
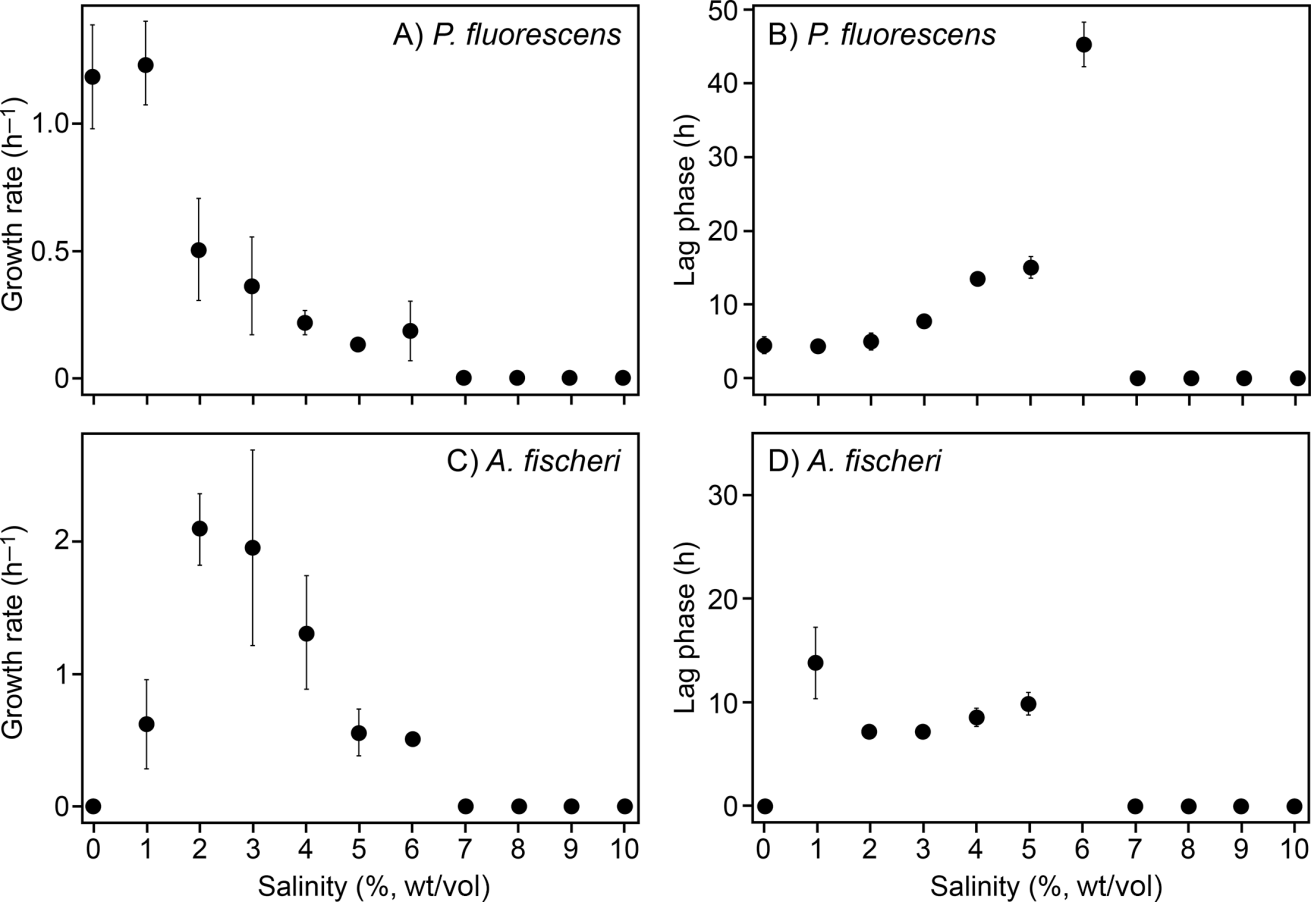
Salinity growth response for aerobic planktonic cultures of *A. fischeri* and *P. fluorescens*. The growth response is depicted for *P. fluorescens* as the growth rate constant (**A**) and the duration of the lag phase (**B**). Similarly, the growth response is depicted for *A. fischeri* as the growth rate constant (**C**) and the duration of the lag phase (**D**). Data points represent the averages of six independent cultures. Error bars show standard deviation.

Growth at different salinities was assessed by examining both the growth rate and the duration of the lag phase in planktonic batch cultures. *P. fluorescens* grew fastest at 0%–1% salinity, and growth rates subsequently declined at increasing salinity until reaching zero at 7% salinity (Fig. 1). Correspondingly, the lag phase for *P. fluorescens* was shortest at 0%–1% and lengthened with increasing salinity (Fig. 1). This pattern confirms that *P. fluorescens* DSM 50090 is not only halotolerant under the current cultivation conditions, thriving at low salinities, but also capable of growth at up to 6% salinity, as reported previously (43). In contrast, *A. fischeri* grew fastest at 2%–3% salinity, and no measurable growth was detected at 0% and 7%–10% salinity (Fig. 1). Similarly, the lag phase for *A. fischeri* was shortest at 2%–3% salinity and increased at both lower and higher salinities (Fig. 1). This pattern confirms that *A. fischeri* DSM 507 grew as a halophile, requiring saline conditions in agreement with its marine origin and previous reports (44).

### Nutrients can be beneficial or detrimental for surviving osmotic stress

**Fig 2:**
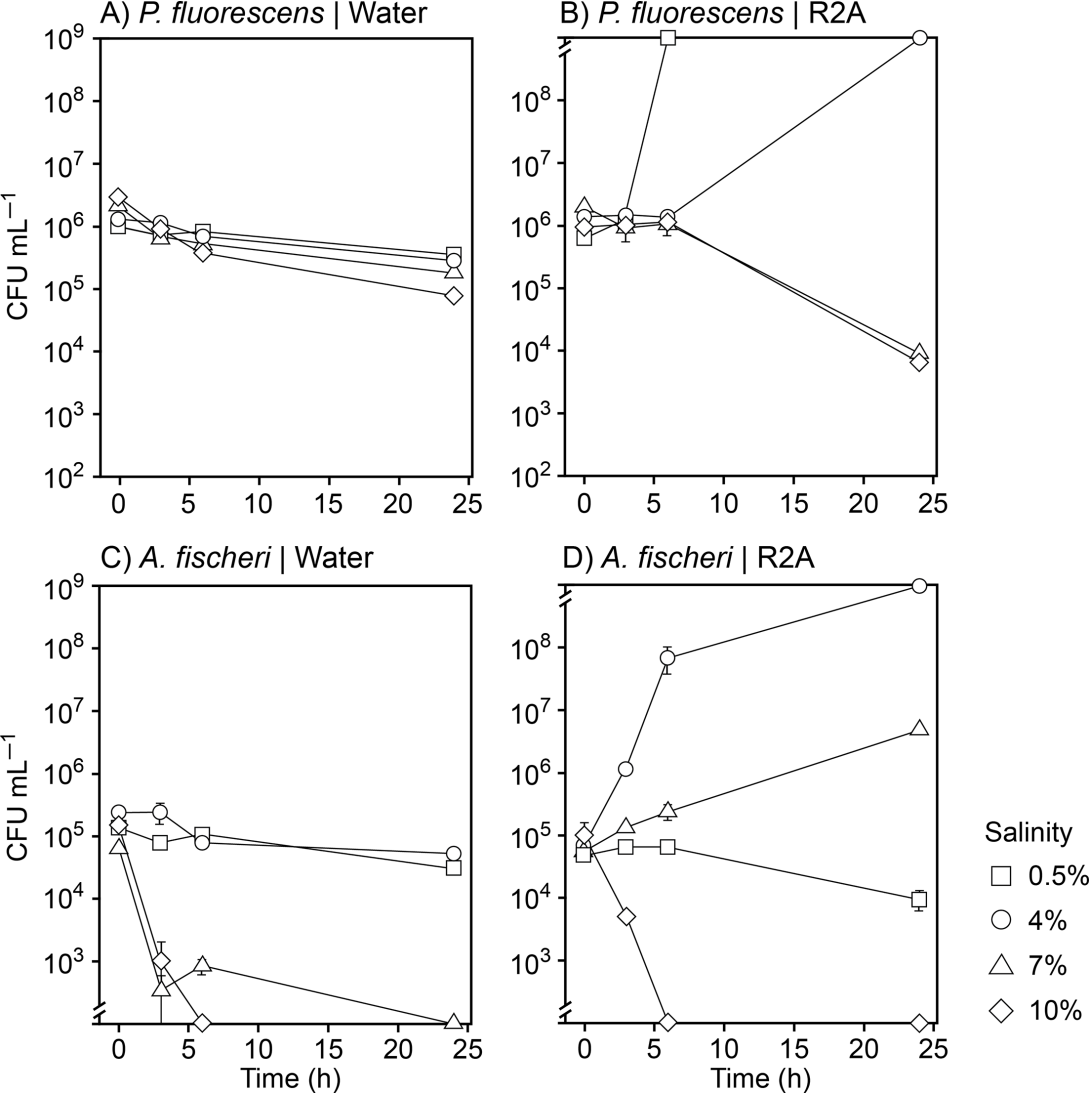
Effect of salinity on survival of planktonic cultures of *P. fluorescens* (**A and B**) and *A. fischeri* (**C and D**). Washed cells were suspended in Milli-Q water (**A and C**) or R2A medium (**B and D**) with 0.5%, 4%, 7%, or 10% (wt/vol) of synthetic sea salt. Cell survival was measured as colony-forming units (CFUs) upon plating on solid R2A medium of the optimum salinity for the culture. Values are averages ± standard deviations (*N* = 3). Data points below the lower break in the *y*-axis reflect no cell survival (detection limit of 5 × 10^2^ CFU mL^−1^). Points above the upper break reflect CFUs too numerous to count (>10^9^ CFU mL^−1^).

While bacterial growth is inhibited at sub-optimal salinities, cells may still survive and resume growth when conditions allow. This would significantly affect the impact of using short periods of osmotic stress to control biofouling. We therefore proceeded to investigate the dynamics of cell death at different salinities by quantifying the concentration of viable *A. fischeri* and *P. fluorescens* cells during exposure to 0.5%, 4%, 7%, and 10% salinity (Fig. 2). *P. fluorescens* survived at all tested salinities when incubated in pure synthetic sea salt solutions. Starting from an initial concentration of approximately 10^6^ CFU mL^−1^, we observed a slight decrease in cell numbers over time irrespective of the salinity (Fig. 2). The most pronounced decrease occurred at 10% salinity, followed by 7% salinity, and so forth. However, the maximum loss of viable cells was less than 1 log (Fig. 2). We repeated the experiment in saline R2A medium to determine if nutrient availability affected survival under osmotic stress. Surprisingly, the loss of viable *P. fluorescens* CFUs increased to 2 log at 7% and 10% salinity. Hence, the cells were more likely to succumb to osmotic stress in the presence of nutrients.

**Fig 3:**
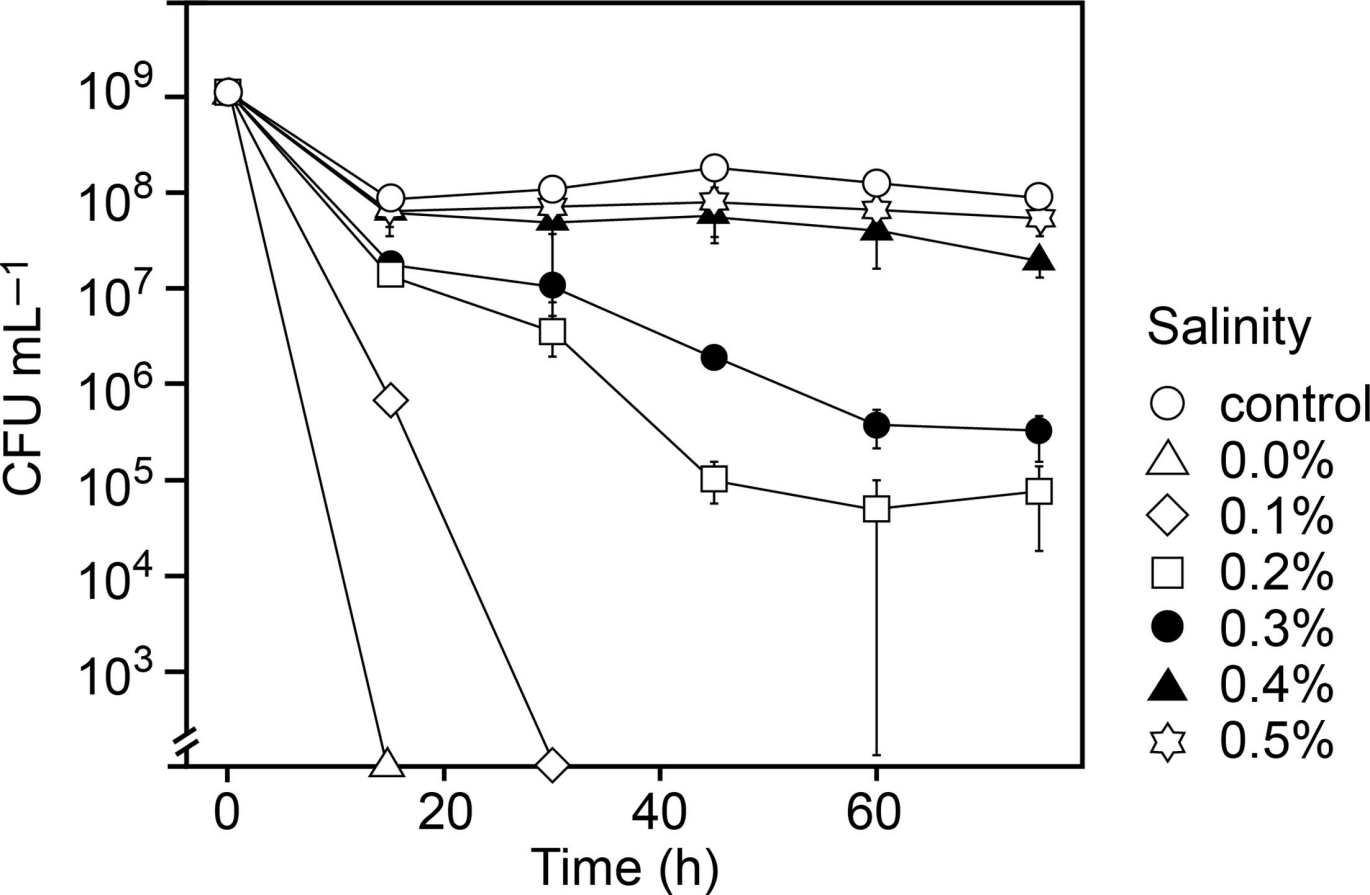
Effect of low salinities on the survival of planktonic cultures of *A. fischeri*. Washed cells were suspended in Milli-Q water with synthetic sea salt in concentrations from 0% to 0.5% and with 2% serving as a positive control. Cell survival was measured as colony-forming units (CFUs) upon plating at 2% salinity solid R2A medium. Values are averages ± standard deviations (*N* = 6). Percent salinity values are wt/vol.

Despite being halophilic, *A. fischeri* was less tolerant of high salinities than *P. fluorescens*, and the CFU concentration decreased to below our detection limit (>3 log reduction) when exposed to 7% and 10% salinity in Milli-Q (Fig. 2). But in contrast to *P. fluorescens*, nutrient availability aided the survival of *A. fischeri*, which maintained viability and even proliferated at 7% salinity in R2A medium, although 10% salinity remained lethal (Fig. 2). *A. fischeri* did not tolerate very low salinities. To understand the boundaries for using low salinity to stress or kill *A. fischeri* and similar halophilic species, we sought to determine the exact salinity at which *A. fischeri* cells die. *A. fischeri* survival was not affected at salinities down to 0.5%. However, the concentration of CFUs declined >3 log at salinities <0.3% (Fig. 3). In particular, salinities of 0.1 or lower were lethal to *A. fischeri* within minutes, while a fraction of cells could withstand salinities down to 0.2% for extended periods (Fig. 3).

### Respiratory activity is not stimulated by osmotic stress

**Fig 4:**
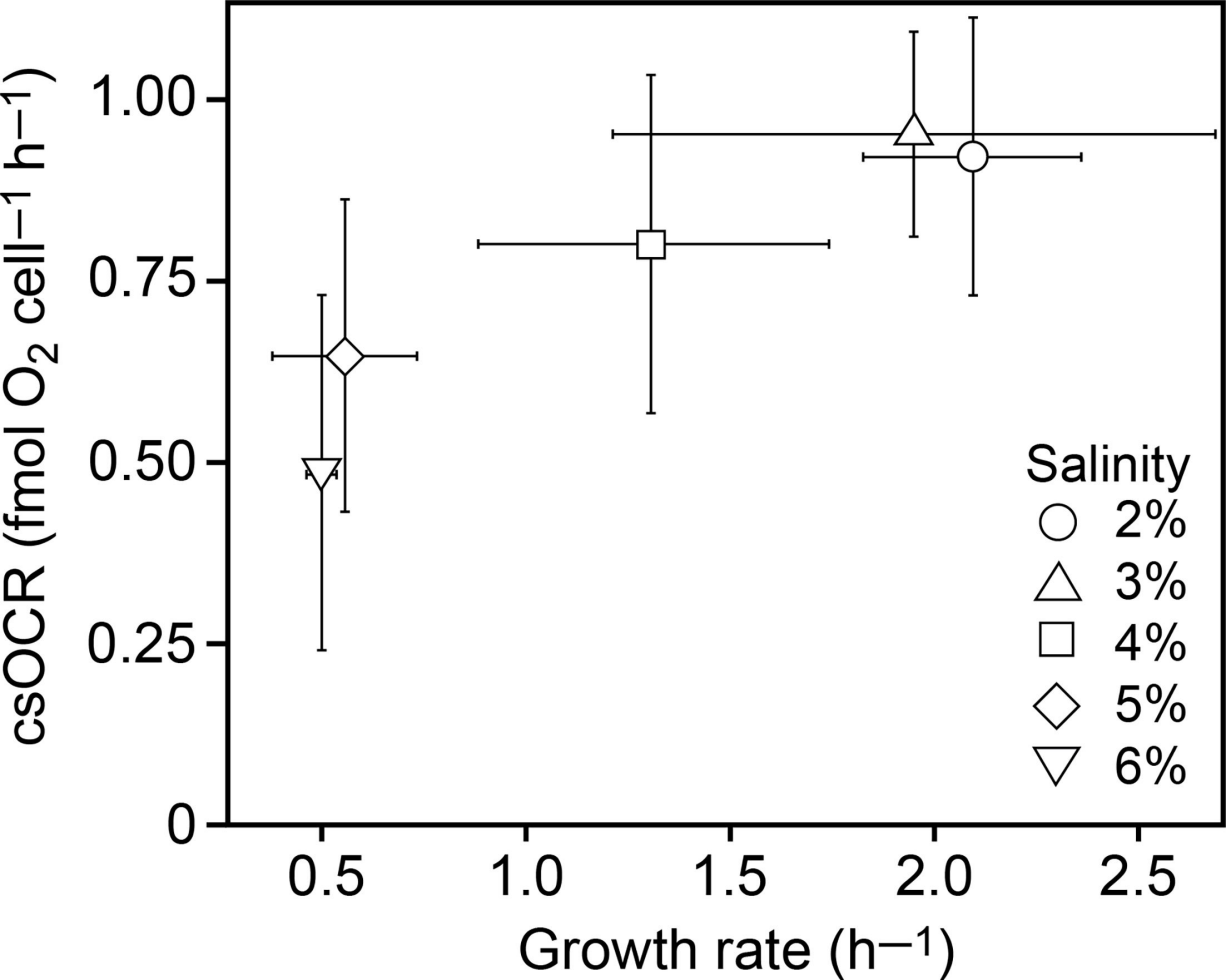
Relationship between the growth rate constant and cell-specific O_2_ consumption rate (csOCR) of *A. fischeri* batch cultures growing exponentially at salinities between 2% and 6% (wt/vol) representing different levels of osmotic stress. Values are averages ± standard deviations (*N* = 5). See Fig. S4 for further details.

Osmoregulation represents an energetic burden for cells, and salt-stressed cells expectedly divert energy from growth processes toward osmoregulation. To examine whether cells needed to increase their metabolic rate to cope with osmotic stress, we measured the csOCR and growth rates for planktonic batch cultures of *A. fischeri* through simultaneous measurements of O_2_ and optical density (OD_600_). The exponentially growing *A. fischeri* had a csOCR of 0.9 fmol O_2_ cell^−1^ h^−1^ at their optimal salinities of 2% to 3%, and the rate decreased gradually with increasing salinity to 0.5 fmol O_2_ cell^−1^ h^−1^ at 6% salinity (Fig. 4; Fig. S4). Thus, during exponential growth, the csOCR of *A. fischeri* correlated with the growth rate (Fig. 4). As expected, the cultures did not grow or consume oxygen at salinity levels of 0% and 7% or higher. Although the cultures grew at 1% salinity, the csOCR was below the detection limit and therefore not shown in the plot.

### Biofilms do not protect *P. fluorescens* and *A. fischeri* from osmotic stress

Biofilms may protect microbial cells against osmotic stress, and fluctuating salinities may, therefore, have less impact on biofouling once a biofilm is established. We, therefore, investigated if biofilms responded differently than planktonic cells to osmotic stress. For this purpose, we grew *A. fischeri* and *P. fluorescens* biofilms under optimal conditions and subsequently assessed the respiratory activity when transferring biofilms to R2A medium with different salinity. The level of O_2_ depletion and time to reach complete depletion were used as proxies for the respiratory activity of the biofilms.

**Fig 5:**
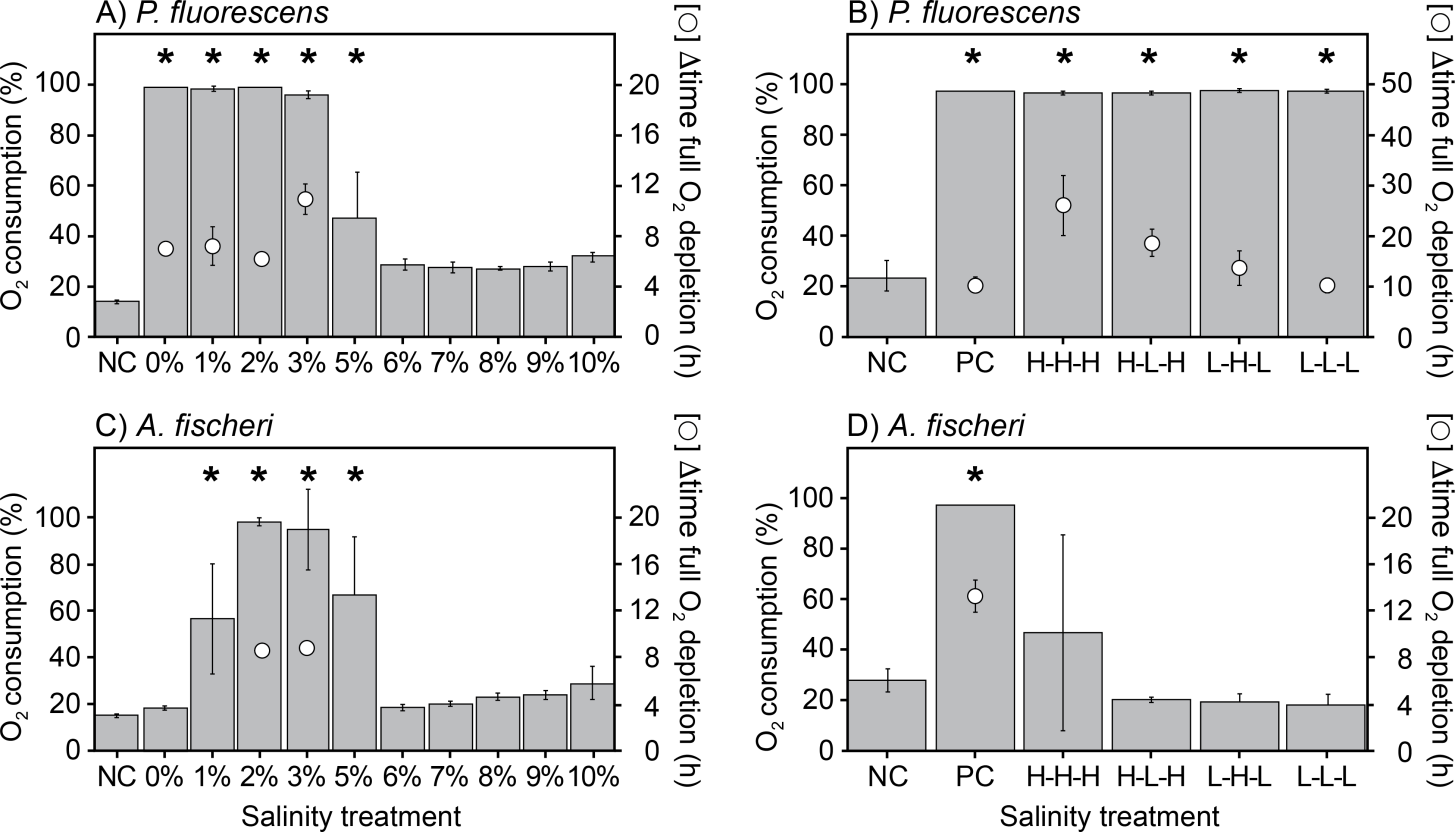
Effect of constant and fluctuating osmotic stress on the O_2_ consumption of pre-grown *P. fluorescens* (**A and B**) and *A. fischeri* (**C and D**) biofilms. The respiratory activity was inferred from the level of oxygen concentration remaining in the well at the end of the incubation. One hundred percent represents full depletion and the incubation time needed to reach this level. (**A and C**) Biofilm O_2_ consumption activity during constant osmotic stress at salinity levels ranging from 0% to 10% (wt/vol) in R2A medium for *P. fluorescens* and *A. fischeri*. (**B and D**) Biofilm O_2_ consumption activity in 2% salinity R2A medium upon fluctuating osmotic stress treatment for *P. fluorescens* and *A. fischeri*. In the latter, pre-grown biofilms were subjected to consecutive 20 min exposures to high (H, 10% [wt/vol]), low (L, 0%), and optimum (positive control [PC], 2% for *A. fischeri* and 0% for *P. fluorescens*) salinity in R2A medium. * indicates a statistically significant difference compared to the negative control (*P* < 0.05). NC: sterile negative control. Values are averages ± standard deviations (*N* = 8). Note the different *y*-axis scales.

Consistent with our data from planktonic cultures, *P. fluorescens* biofilms respired at 0%–5% salinity (Fig. 5), with the highest activity at 0%–2%. At 3% salinity, the biofilms also depleted oxygen from the medium, but it took longer to reach depletion, indicating a slower metabolic rate. Respiration could not be detected at 6%–10% salinity. Similarly, *A. fischeri* biofilms showed maximum respiratory activity at 2%–3% salinity (Fig. 5). The activity was lower at 1% and 5% salinity, and no oxygen consumption was observed at 0% or at salinities of 6% or higher (Fig. 5). Thus, biofilm formation did not protect the bacteria from osmotic stress.

If the osmotic stress is temporary, as it would be in a batch RO filtration unit, biofilms might recover and continue to grow during periods of optimal salinity. We, therefore, assessed the metabolic activity of biofilms as they recovered (in optimal growth media) after a short series of fluctuating salinity (0% and 10%, 20 min in each solution). *P. fluorescens* biofilms were not killed and recovered from all the treatments. However, the longer it was exposed to high (10%) salinity, the longer the recovery time was, as indicated by the time required to deplete oxygen from the growth medium during recovery (Fig. 5). After 3 × 20 min at high salinity, it took approx. 15 h longer to reach oxygen depletion. This delay indicates that cells were stressed or killed by the change in salinity, but the population could recover and regrow when given sufficient time. In contrast, fluctuating osmotic stress severely affected the respiratory activity of *A. fischeri* biofilms (Fig. 5). Respiratory activity was only detected in biofilms continuously exposed to high salinity, indicating that any exposure to low salinity (0%) killed the bacteria (Fig. 5). These results are also consistent with how planktonic *P. fluorescens* and *A. fischeri* respond to osmotic stress.

## DISCUSSION

This study examined the impact of constant and fluctuating hypo- and hyperosmotic stress on survival and activity of both planktonic and biofilm cultures of *P. fluorescens* and *A. fischeri*. We, hereby, aimed to determine if fluctuating salinity can be used as an effective measure to limit microbial activity and growth in batch RO or similar systems.

### Hypo- and hyperosmotic stress can eradicate planktonic cells

We first addressed if the halotolerant bacterium *P. fluorescens* and the halophile *A. fischeri* could survive at salinities outside of their growth-permissive salinity range. Planktonic cultures of *P. fluorescens* and *A. fischeri* were killed at salinities outside this range, with species-specific loss of viability influenced by exposure time and nutrient availability (Fig. 2 and 3). As noted in the Introduction, osmoadaptation is energetically costly, and the ability to withstand salinity stress likely depends on environmental nutrient availability, both as an energy source and as a source of precursors for compatible solutes (8). Our observations for *P. fluorescens* exposed to constant salinity stress do not agree with this assumption. When challenged by 7% and 10% salinity in nutrient-rich R2A medium, all *P. fluorescens* cells survived the initial 6 h, but thereafter, 99% of cells lost viability (Fig. 2). In contrast, 10-fold more cells survived during long-term exposure to 7% and 10% salinity in the absence of nutrients. These observations can be explained if the absence of nutrients drives *P. fluorescens* cells into a dormant state, which is part of a general stress response in *Pseudomonas* that promotes survival under extreme environmental conditions (23, 45). Such a state likely involves processes beyond a simple metabolic shutdown (46). Consistent with this interpretation, our biofilm experiments (Fig. 5) demonstrate that neither *P. fluorescens* nor *A. fischeri* respire oxygen at detectable levels when incubated at salinities outside their growth range. In contrast to *P. fluorescens*, *A. fischeri* tolerated hyperosmotic stress under nutrient-rich but not nutrient-poor conditions (Fig. 2). In fact, *A. fischeri* survived exposure to 7% salinity in the R2A medium, while cells rapidly lost viability at this salinity in the pure salt solution. The R2A medium contains compounds that may serve as compatible solutes or their precursors (47), which *A. fischeri* can take up from the environment (25) and thereby facilitate osmoadaptation. Indeed, marine *Vibrio* species can rapidly import exogenous osmolytes through high-affinity transport systems (48). Notably, weak growth of *A. fischeri* was observed at 7% salinity in R2A medium in the experiment where growth was quantified by CFU counts (Fig. 2). This level of growth was likely undetectable in the experiments where growth was quantified by optical density (Fig. 1). Similar to our observations in *A. fischeri*, the growth and survival of budding yeast are more severely compromised by periodic osmotic stress under glucose-poor conditions than under glucose-rich conditions (14). This is likely because yeast cells are unable to initiate their specific osmotic stress response when deprived of their primary energy source and the precursor required for compatible solute synthesis (14).

The experimental design of the salinity stress survival assay (Fig. 2) involves subjecting cells to a hypoosmotic shock when they are transferred from the high-salinity solutions to the lower-salinity media used for CFU-based determination of viability. However, because survival was high in all *T* = 0 samples (Fig. 2), in which cells were likewise exposed to the same hypoosmotic shock, we consider this effect to be modest. The hyperosmotic stress-induced eradication of *P. fluorescens* and *A. fischeri* was more or less salinity-dose independent under conditions where the salinity exceeded their permissive range for growth (Fig. 2). This is important because it can help define limits of salinity fluctuations needed for efficient biofouling control in batch RO systems. It should be noted that our use of synthetic sea salt with a mixed ionic composition to adjust salinity of growth media did not affect the growth response of the two species as compared to previous studies where salinity was adjusted with sodium chloride (43, 49). Sodium has an eightfold higher molar abundance than the second most abundant cation, magnesium, in the synthetic sea salt. Although magnesium chloride can exert strong osmotic stress (50), its effect on cell growth is apparently negligible compared to that of sodium chloride at the sodium-magnesium ratio of seawater.

Halophilic bacteria are sensitive to both hyper- and hypoosmotic stress. Halophiles generally respond to hypoosmotic shock by release of cytoplasmic metabolites through mechanosensitive channels in the cell envelope (51). The initiation and termination of this osmotic response depend on different channels opening and closing at different membrane tensions. *A. fischeri* expresses at least two mechanosensitive channels under both high and low salinity conditions (25). We observed that *A. fischeri* cells growing at 2% salinity survived hypoosmotic shock upon transfer to 0.4% and 0.5% salinity, while survival was immediately reduced when challenged by 0.2% and 0.3% salinity and survival was minimal at 0% and 0.1% salinity (Fig. 3). Unlike its response to hyperosmotic stress, *A. fischeri,* thus, shows a clear dose-dependence in its ability to survive hypoosmotic stress, and even small changes in salinity below its permissive range for growth can have a strong impact on cell survival (Fig. 3). These results suggest that very high salt concentrations are required to efficiently kill cells by short-term hyperosmotic stress; however, hypoosmotic shock, as could be achieved by flushing RO membranes with permeate, can serve as the basis for instantly eradicating halophilic members of fouling communities.

### Cells respire slower and increase maintenance energy requirements during hyperosmotic stress

Growth at hyperosmotic salinities is energetically demanding and increases the maintenance energy requirement of cells (9–12). We aimed to determine if cells compensate for this energy demand by increasing their respiratory activity and thereby sustain growth over a wider range of salinities. Addressing this question is important for predicting if nutrient-limited cells are less tolerant to hyperosmotic stress than cells growing under nutrient-rich conditions. We, therefore, developed an experimental setup to measure highly replicated csOCR during exponential growth of *A. fischeri* by concurrent measurements of optical density (OD) and oxygen concentrations. Within the tested salinity range of 2% to 6%, the csOCR was positively correlated with the growth rate of cultures (Fig. 4), and the csOCR was not increased in response to osmotic stress. This result is consistent with observations made for natural microbial communities in marine waters (12).

The hyperosmotic salinity stress negatively affected cell growth proportionately more than the csOCR because the growth rate decreased by approximately 80%, while csOCR decreased by 50% within the tested salinity range. This likely reflects elevated maintenance energy requirements associated with a reallocation of resources from growth to osmoadaptation, stress response, and repair processes. While cells can compensate for nutrient limitation by slowing down or arresting growth, maintenance energy requirements must be met to sustain cell integrity (52). The higher proportion of the energy budget allocated to maintenance functions of cells under hyperosmotic stress likely makes them vulnerable to eradication if they concurrently are challenged by nutrient starvation.

### Biofilms are sensitive to osmotic stress, and salinity fluctuations may limit their formation and growth

Because biofilm formation can enhance tolerance to osmotic stress, we assessed whether biofilm cultures of *P. fluorescens* and *A. fischeri* differed in salinity tolerance from their planktonic counterparts. OCR of pre-grown biofilms was measured after transfer to R2A medium with varying salinities (Fig. 5). The salinity dependence of biofilm respiratory activity paralleled the growth of planktonic cultures (Fig. 1), with activity detected only within the salinity range permissive for planktonic growth (Fig. 5). Our experiments thus suggest that biofilm formation does not protect *P. fluorescens* and *A. fischeri* cells from osmotic stress. However, we cannot exclude that experiments with thicker or denser biofilms than those that develop on PEG lids would yield different results.

The *P. fluorescens* and *A. fischeri* biofilm cultures were used to assess whether hypo- or hyperosmotic stress during salinity fluctuations is more detrimental than constant osmotic stress. *A. fischeri* biofilms showed reduced OCR following short-term (20 min) fluctuations between 0% and 10% salinity (Fig. 5), consistent with observations for planktonic *A. fischeri* cultures (Fig. 3). Repeated short-term exposures to 10% salinity also decreased biofilm OCR (Fig. 5). Considering that planktonic *A. fischeri* cultures die when exposed to salinities exceeding their upper growth limit (Fig. 2), it is likely that continuous salinity fluctuations reaching this salinity level would completely eradicate the biofilm. *P. fluorescens* biofilms efficiently survived fluctuating salinity stress and maintained a level of respiratory activity regardless of the fluctuation regime (Fig. 5). However, the respiratory activity was negatively correlated with the number of exposures to 10% salinity (Fig. 5), and repeated exposure to such hyperosmotic condition would likely eradicate the biofilm. In agreement, a previous study (53) demonstrated that *P. fluorescens* biofilms were killed during multiple exposures to high (9%) salinity.

### Implications for biofouling control in RO systems

In RO systems, feedwater salinity ultimately determines the maximum brine salinity (54). Only RO systems treating saline water can achieve brine salinities in the percent range, which may inhibit bacterial growth and survival. However, our results suggest that batch-mode operation with oscillating brine salinity in such RO systems has the potential to reduce biofouling. The microbial communities responsible for membrane fouling in RO systems are diverse, comprising members with differing salinity optima (55). Effective biofouling control, therefore, requires identifying the optimal salinity range and exposure duration for oscillations. In this context, the findings that hyperosmotic stress becomes lethal to cells once salinities exceed their upper growth limit and that halophilic bacteria are instantly killed when exposed to 0% salinity (as could be achieved through permeate backflushing) are particularly significant. Continuous salinity oscillations may not only mitigate existing biofouling but also delay or prevent biofilm formation. Achieving these outcomes requires designing oscillations to induce sufficient osmotic stress at a frequency that prevents bacterial populations from recovering between cycles. Furthermore, water pretreatment procedures typically create nutrient-limited conditions in most RO systems (56). Our findings suggest that such nutrient limitation reduces the capacity of cells for continuous osmoadaptation, thereby enhancing the effectiveness of salinity oscillations in biofouling control.

## SUPPLEMENTAL MATERIAL

Supplemental material aem.02411-25-s0001.pdf Figures S1 to S4.

ASM does not own the copyrights to Supplemental Material that may be linked to, or accessed through, an article. The authors have granted ASM a non-exclusive, world-wide license to publish the Supplemental Material files. Please contact the corresponding author directly for reuse.
