# Supplementary material for: Limits of bacterial osmoadaptation during planktonic and biofilm growth: a step toward effective biofouling control
Source: Appl Environ Microbiol. 2026 Apr 21;92(5):e02411-25. doi: 10.1128/aem.02411-25 (PMC13188924; doi:10.1128/aem.02411-25)
Supplement: Supplemental material — Figures S1 to S4. [file aem.02411-25-s0001.pdf]

SUPPLEMENTARY MATERIAL

Supplementary Figures S1-S4

Limits of Bacterial Osmoadaptation During Planktonic and Biofilm Growth: A Step Towards Effective Biofouling Control

Jan Struckmann Poulsen[a*], Arya Van Alin[b], Peter Bundgaard Larsen[a], Freja Marie Nordby Haarder[a], Rikke Louise Meyer[a,b], Klaus Koren[a], Kasper Urup Kjeldsen[a]

[a] Department of Biology, Section for Microbiology, Aarhus University, Ny Munkegade 114, 8000 Aarhus C, Denmark

[b] Interdisciplinary Nanoscience Centre (iNANO), Aarhus University, Gustaw Wieds Vej 14, 8000 Aarhus C, Denmark

*Present address: Jan S. Poulsen. Danish Technological Institute, Kongsvang Allé 29, 8000 Aarhus C, Denmark

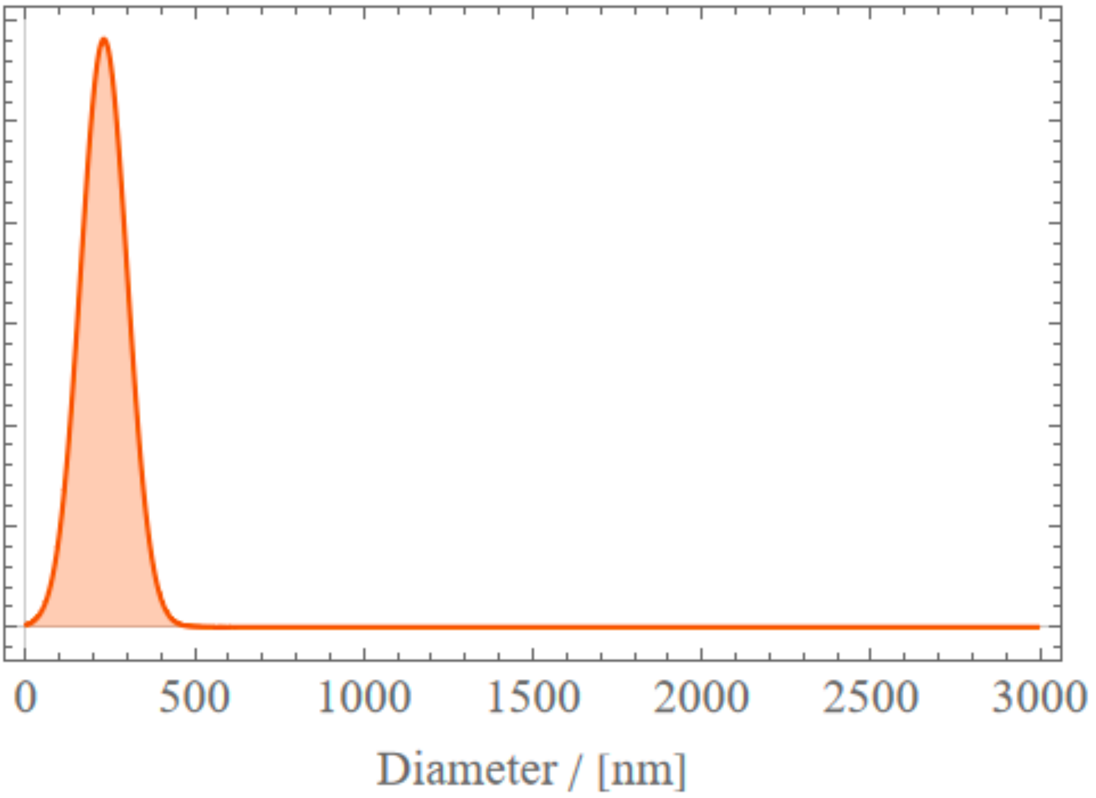

Figure S1. Size distribution of the oxygen-sensitive nanoparticles. The size was characterized by dynamic light scattering (see main text for details). The y-axis shows particle count on an arbitrary scale.

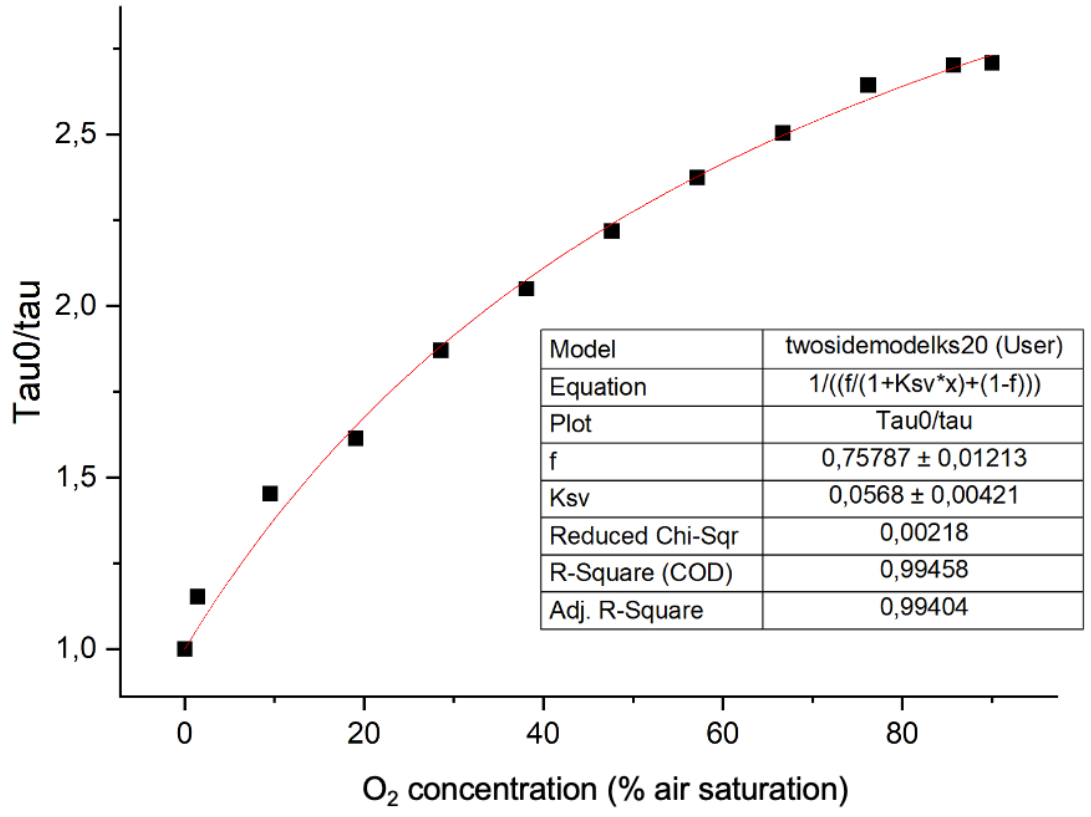

Figure S2. Multipoint calibration curve of oxygen-sensing nanoparticles. The calibration curve was constructed with the Stern-Volmer relationship that relates the change in luminescence intensity and luminescence lifetime (Tau0/tau) to the O_2 concentration. The model fitted to the measured data is shown as a solid red line. All measurements were performed at room temperature.

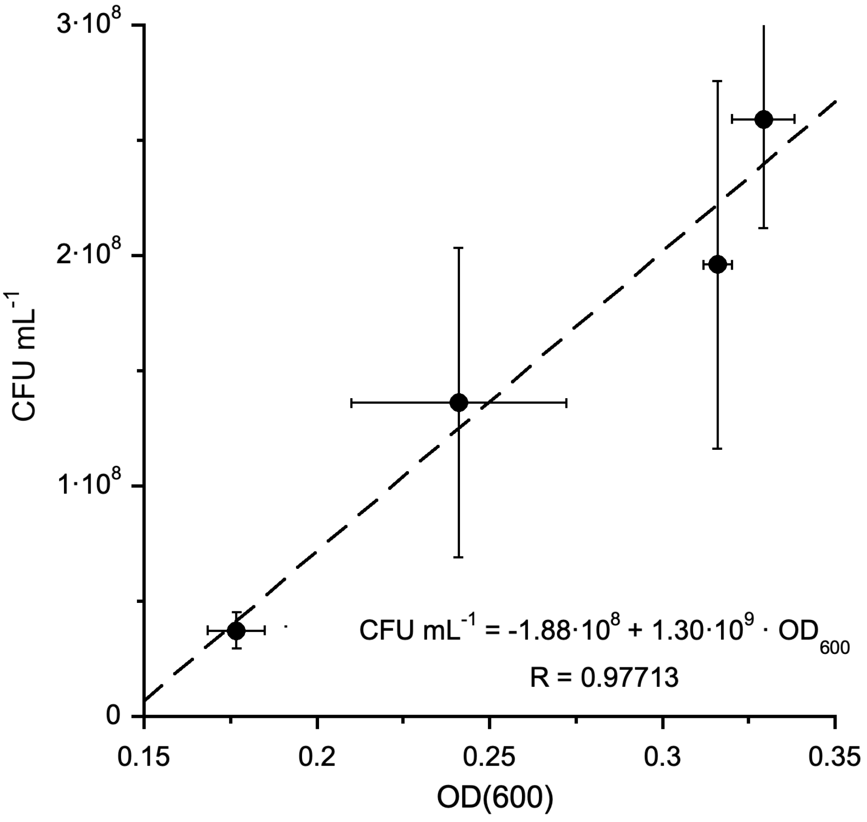

Figure S3. Correlation between viable cell counts (CFU/mL) and optical density (OD_{600}) for *A. fischeri* planktonic cultures. The linear regression (dotted line) was used to convert OD_{600} measurements into viable cell counts in the cell-specific oxygen-consumption rate experiment. Values are averages ± standard deviation (n = 3).

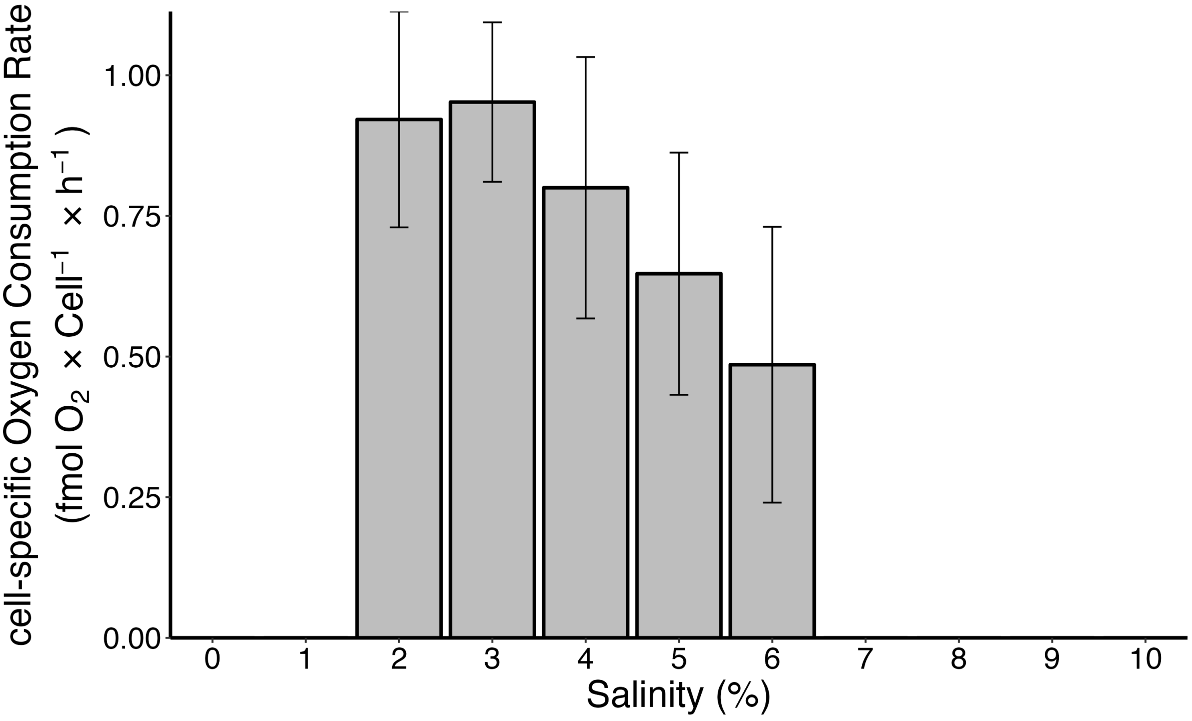

Figure S4. Cell-specific oxygen consumption cate (csOCR) of exponential phase of planktonic *A. fischeri* cultures grown at salinities ranging from 0 to 10%. The csOCR was derived by simultaneously measuring dissolved oxygen concentrations and optical density of the cultures. No growth and no O_2 consumption were observed at 0, 7, 8, 9 and 10% salinity. Growth was observed at 1% salinity, but the csOCR was too low to be reliably determined. The optical density of cultures was converted to cell density via the calibration curve shown in supplementary Figure S3. Values are averages ± standard deviation (n = 5).
